# A Mobile Health Behavior Intervention to Reduce Pain and Improve Health in Older Adults With Obesity and Chronic Pain: The MORPH Pilot Trial

**DOI:** 10.3389/fdgth.2020.598456

**Published:** 2020-12-18

**Authors:** Jason Fanning, Amber K. Brooks, Edward Ip, Barbara J. Nicklas, W. Jack Rejeski, Beverly Nesbit, Sherri Ford

**Affiliations:** 1 Department of Health and Exercise Science, Wake Forest University, Winston-Salem, NC, United States; 2 Department of Anesthesiology, Wake Forest School of Medicine, Winston-Salem, NC, United States; 3 Department of Biostatistical Sciences, Wake Forest School of Medicine, Winston-Salem, NC, United States; 4 Department of Internal Medicine, Wake Forest School of Medicine, Winston-Salem, NC, United States

**Keywords:** smartphone, design, mHealth, physical activity, older adults, pain, sedentary behavior, wearable device

## Abstract

Chronic, multisite pain is a common phenomenon in aging and is associated with a host of negative health outcomes. It is a complex and multifaceted condition that may be exacerbated by weight gain and long periods of inactivity. Unfortunately, older adults suffering from chronic pain have unique barriers limiting access to center-based behavior change interventions. The MORPH study first adapted and iteratively refined an evidence-based group-mediated intervention for delivery in the home via mHealth tools (a smartphone app, teleconferencing software, wearable activity monitor, smart weight scale). This was followed by a pilot randomized controlled trial (RCT) meant to assess feasibility of the MORPH intervention, and to examine initial effects on physical function, pain, weight, and sedentary behavior. We recruited low-active and obese older adults with multisite pain to partake in a series of N-of-1 refinement studies (*N* = 5 total) or a 12-week pilot RCT delivered largely in the home (*N* = 28 assigned to active intervention or wait-list control). The refinement phase identified several key technological (e.g., selection of a new smart weight scale) and user interface (e.g., clarification of in-app phrasing) modifications that were made before initiating the RCT phase. Analyses of covariance, controlling for baseline values, sex, and age indicated effects favoring the intervention across all domains of interest: there was a substantially clinically meaningful difference in short physical performance battery scores (0.63 points, *η*^2^ = 0.08), a moderate-to-large difference in PROMIS pain intensity scores (5.52 points, *η*^2^ = 0.12), a large difference in body weight (2.90 kg, *η*^2^ = 0.207), and a moderate effect on adjusted ActivPAL-assessed sedentary time (64.90 min, *η*^2^ = 0.07) with a small effect on steps (297.7 steps, *η*^2^ = 0.01). These results suggest a largely-home delivered movement and weight loss program for older adults with pain is feasible and recommendations are provided for future programs of this nature.

**Clinical Trial Registration:**

ClinicalTrials.gov, Identifier: NCT03377634.

## INTRODUCTION

Chronic pain is common in older adults (1) and is associated with reduced mobility, obesity, elevated risk for falls, depression and anxiety, and social isolation (2, 3). Unfortunately, many of these negative health outcomes operate cyclically with chronic pain. For instance, musculoskeletal strain and inflammation associated with obesity increase pain symptoms, while the experience of pain can contribute to obesogenic emotional eating (2). Similarly, while participating in movement can help to manage pain symptoms and prolonged sitting can contribute to stiffness and inflammation that worsens symptoms, pain itself acts as a potent barrier to movement, contributing to further pain and inactivity (2-4). Unfortunately, the viability of center-based health behavior interventions for this subgroup of older adults is compromised by the combined effects of social isolation and limited transport options (5); yet older adults suffering from chronic pain are likely to require a high degree of ongoing social and behavioral support to make meaningful and lasting behavior change and this necessitates the use of remote methods of delivery (6,7). To this end, we developed the Mobile Intervention to Reduce Pain and improve Health (MOPRH) study (8) wherein we followed a user-centered design philosophy to iteratively develop a home-based mHealth-supported activity promotion and weight reduction protocol founded in social cognitive theory (6), self-determination theory (7), and principals of group dynamics (9). Herein, we present the results from MORPH, which included a small user-centered design phase followed by a 12-week randomized controlled pilot study evaluating the effects of the intervention on pain and functional health.

Successfully modifying challenging behaviors such as diet and physical activity—especially under the influence of chronic pain—requires a program that (a) instills self-regulatory skills and self-efficacy for movement and weight loss, (b) is delivered primarily in the home, and (c) facilitates social connection between participants and intervention staff. Retaining a social structure in the home recognizes one of three pillars of self-determination theory—that human well-being requires feeling a sense of deep connection and relatedness with others (7)—and is represented in most key constructs in social cognitive theory [e.g., the effect of modeling and verbal persuasion on self-efficacy, social outcome expectencies (6)]. In our own work promoting weight loss and physical activity, we employ a technology-supported group-mediated model, leveraging group structure as a tool of behavior change where individuals raise and address barriers, act as accountability agents, and more (10-14). We augment weekly group meetings with daily smartphone support tools to cue mastery experiences, enhance self-regulation, and provide ongoing connection to the research team and group members (8, 13, 14).

Unfortunately, many effective activity-promotion interventions for older adults require regular attendance at a community or research center, which limits scalability and sustainability. As a consequence, a very small number of older adults have access to such programs, an effect that is further exacerbate by chronic pain, obesity, and transportation barriers. Moreover, extant group-based behavioral interventions are designed for delivery over a finite period. Thus, completion of treatment is marked by removal of key sources of behavior change and maintenance of change—social connection and monitoring/support for self-regulation. Therefore, the MORPH study first developed and then piloted a home-based protocol that leverages widely used technologies that can be scaled broadly and feasibly delivered long-term.

Success in adapting effective user-centered, socially mediated interventions for home delivery requires careful iterative development conducted hand-in-hand with members of the target population. In the spirit of contemporary engineering-inspired design frameworks such as the multiphase optimization strategy [i.e., MOST (15)]. MORPH was designed as a series of iterative and progressive studies meant to identify useful intervention components while quickly isolating and removing design elements that were either ineffective or a nuisance. This was accomplished in two phases: in phase 1 we conducted a series of N-of-1 studies meant to identify and address technical and usability issues. Phase 2 comprised a separate randomized controlled pilot study to evaluate the potential efficacy of the full 12-week intervention and to identify additional usability and technical issues. The purpose of this paper is to briefly note the findings from the iterative refinement phase as a guide to future older adult-oriented app development, and then provide the results of the MORPH pilot study. Because MORPH itself is an iteration upon a center-based, mHealth-driven lifestyle intervention (14), we expected iterative testing to quickly identify and address usability issues in a small number of iterations. Regarding phase 2, we hypothesized that participation in MORPH was feasible for older adults with chronic pain and that it would result in reductions in self-reported pain, improvements on the short physical performance battery [SPPB (16)] score, reductions in body weight, and improvements in daily steps and sedentary time relative to a wait-list control group.

## MATERIALS AND METHODS

### Participants

We recruited older adults with chronic multisite pain to participate in the MORPH study. Participants were recruited for the two phases of the study between March 2018 and October 2019. The methods have been described in detail elsewhere (8). In brief, eligible participants were aged 55–85 years of age with a body mass index (BMI) of 30–45 kg/m^2^. They were low active (i.e., did not participate in regular resistance training and/or more than 20 min of aerobic exercise on 2 or more days per week in the previous 6 months) and weight stable (i.e., had not lost or gained more than 5% of their body weight in the previous 6 months), and had no contraindication to exercise. Participants were also required to own a personal smartphone and had pain in at least two of the following sites on most days in the previous 3 months: back, neck, shoulders, hips, or knees. Those who participated in the first phase of the study could not participate in the second phase. Participants were recruited through existing study databases, targeted mailings, and pamphlets/flyers placed throughout clinics in Winston-Salem, NC. The study protocol was approved by the institutional review board at Wake Forest School of Medicine, all participants signed an approved informed consent document, and the trial was registered at ClinicalTrials.gov (NCT03377634).

### Phase 1: Refinement

**FIGURE 1 ∣:**
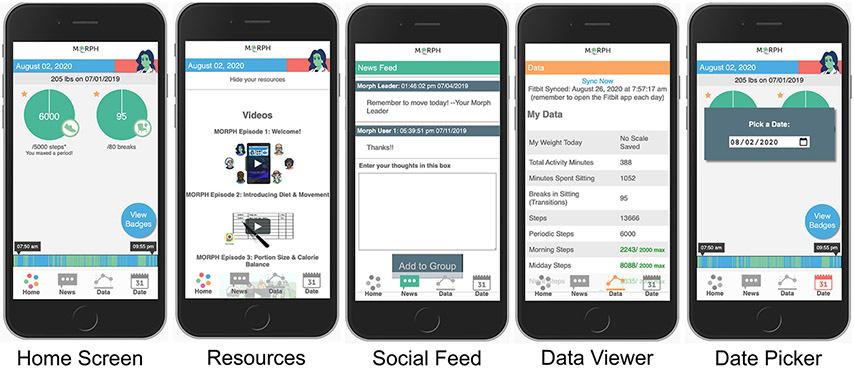
Example MORPH screens. The first depicts the landing page, with quick-view stepping and break information, a daily weigh-in display, access to mastery badges, and the activity timeline bar wherein green represents movement and blue represents non-movement. The second depicts the in-app social feed. The third depicts the daily data summary page, which displays data from the smart scale and the wearable activity monitor. The final displays the resources page, where participants can view weekly videos and listen to weekly podcasts.

Participants were recruited one at a time to participate in one lab visit and a 1-week field test. The primary aim of the design phase was to gather pragmatic feedback from members of the target population on the app’s form and function prior to its use in the randomized controlled trial phase. Participants first completed an in-person semi-structured interview wherein they received the Fitbit Alta device, a smart weight scale, and the study Companion app (detailed below) prepopulated with user data (e.g., step data, participant communications) for the purposes of testing. The participant was instructed to guide the researcher through all features of the app, and that the research team member would simply listen, only providing feedback if the participant became stuck. The participant was instructed to *Think Aloud* (17, 18), or narrate their inner thoughts, attempting to speak continuously. During this *Think Aloud* procedure, the research team member would prompt the participant to continue to think aloud if they fell quiet. They aimed to have the participant interact with each of the 15 focal areas (see Supplementary Table 1) and Core screens (see Figure 1). The researcher noted when any feature was incorrectly described, if the participant later correctly amended their description, and any time an interaction with a feature was non-intuitive. Once the participant interacted with all core app elements, the interviewer debriefed the participant, describing each core feature and its purpose, allowing the participant to once again interact with each element. Participants were then signed into their own accounts on their personal device, oriented to the importance of moving across the day as it relates to pain, and taught to weigh daily and to view the app frequently throughout the day to become familiar with their own movement patterns.

For 1 week following this appointment, participants engaged with the app in their home. They were asked to make note of any technical issues encountered during this period. On completion of the in-home testing week, participants returned to the research center to discuss their experiences. They submitted any technical issues and completed the System Usability Scale (19). Upon completion of this appointment, the participant’s feedback was discussed among the study team to identify features to modify. All technical errors were addressed immediately. Aesthetic recommendations were immediately implemented if they involved erroneous formatting or overtly unclear structure or phrasing. Non-critical recommendations were prototyped within the app and displayed to subsequent participants in unmodified and modified forms, allowing the participant to select their preferred option. This process was repeated until no substantive revisions were required.

### Phase 2: Pilot Randomization and Follow-Up

Participants were randomized using a web-based randomization scheme developed by the study biostatistician in two waves to either the active intervention or wait-list control group. Randomization was conducted following baseline testing and was stratified by sex with random block sizes. Participant follow-up visits were scheduled at 12 weeks. The primary aim for this phase was to determine the feasibility of the mHealth-supported MORPH intervention among older adults with chronic multisite pain. We aimed to recruit between 24 and 30 participants to sufficiently test study procedures and to provide useful estimates for a future two-arm randomized trial while staying within budget and resource constraints (20-22).

### Intervention

Eligible participants completed one assessment appointment prior to the start of the intervention. During this appointment, participants completed questionnaires, anthropometric, and physical function assessments. Participants also received the ActivPAL™ 4 activity monitor. We selected the ActivPAL to suit our focus on moving to reduce sitting time. The ActivPAL is capable of accurately distinguishing posture and as such is regarded as the gold standard for assessing sedentary behavior (23). Next, those randomized to receive the intervention attended a pre-study orientation. Here, participants met with the behavioral interventionist for orientation on the MORPH Companion App (described below), and the use of the Fitbit Alta wearable activity monitor. The Alta was selected as wrist-worn devices are often more acceptable and produce better long-term compliance, which are key features in a feedback device (24). Additionally, given that all device data are stored perpetually on Fitbit servers, we selected a device that is not capable of collecting sensitive heart rate and global positioning data. In addition to the Fitbit, participants received a BodyTrace cellular-enabled weight scale. This device is equipped with a cellular chip and is pre-registered to post data to a research server. Participants were asked to weigh approximately daily throughout the program, and these weights were made available immediately to the research team via the MORPH Portal and to the participant within their MORPH Companion App (each described below). Finally, participants were introduced to the use of the Webex teleconferencing software suite. Webex was selected for use in MORPH as it is institutionally supported in our health system. Finally, while the interventionist added the study applications to the participant’s device, the study nutritionist provided orientation on food tracking.

During the first 3 weeks of the intervention, participants met in-person once weekly in small groups led by a behavioral interventionist and nutritionist. These group sessions integrated elements from effective in-person and remote social-cognitive weight loss and exercise trials (13, 25), web-based pain and coping skills training (26), and mindfulness-based relapse prevention (27). The purpose of these appointments was to gain foundational knowledge on dietary behavior change, the importance of movement during pain, the influence of changing pain symptoms on weight and diet behavior, and the value of mindfulness for recognizing and interrupting these dynamic barriers (27). They also allowed for the development of relationships between participants and group leaders. Based on lessons learned during MORPH phase 1, we also included one additional weekly call between each participant and the behavioral interventionist during the first 3 weeks to practice use of the Webex software. During weeks 4–12, participants met as a group in a private video conferencing room. These meetings enabled the behavioral interventionist and nutritionist to provide didactic content related to dietary behavior change, increasing physical activity throughout the day, pain management, and mindfulness. All group sessions included participant-led conversations on successes over the previous week. Participants also discussed barriers and brainstormed strategies for overcoming these barriers. Each session included a brief period for guided mindful skill practice.

Throughout each week, participants engaged in several self-monitoring practices. For instance, participants were instructed to wear the Fitbit activity monitor daily and to remove the device only for charging. The MORPH software suite leveraged the Fitbit application programming interface (API) to collect minute-level activity data throughout the day. These data were then processed in real time and summarized in visual and numeric feedback that were available to both the researcher via a MORPH portal, and the participant via the MORPH Companion smartphone app, both of which were developed by the study Co-PI (JF). Within the MORPH researcher portal, interventionists could view participants’ daily “timeline” bars, which displayed patterns of movement across the day such that inactive minutes were displayed in blue and active minutes in green. This same timeline bar was available in real-time within the participant’s MORPH app (see Figure 1; described in depth below). Additionally, researchers could view daily minutes spent moving, daily steps, and daily “breaks” (i.e., number of transitions between stepping and non-stepping minutes). The behavioral interventionist used these data to tailor participant goals each week, and over the course of 12 weeks participants generally worked toward a maximal maintenance goal of 10,000 daily steps and 100 daily breaks. Participants also maintained daily dietary logs. The nutritionist leveraged these data to tailor participant caloric restriction goals, where participants generally aimed to achieve an approximate 400 kcal deficit from daily weight maintenance requirements, with a minimum caloric intake goal of 1,100 kcal/d for women and 1,200 kcal/d for men.

Throughout the program, intervention participants were asked to monitor their smartphone app—the MORPH Companion App (Figure 1)—at least once daily but were encouraged to engage with the app frequently throughout the day. There were two main goals of this Companion App. First, while participants aimed to achieve an overall daily step goal, they were encouraged to prioritize *frequent* bouts of movement throughout the day above and beyond singular exercise bouts in an effort to minimize sustained periods of sitting. We attempted to encourage day-long movement by providing specialized feedback and incentives within the Companion App. Participant physical activity was displayed on their timeline bar. Participants aimed to distribute green bouts of physical activity evenly throughout the day (i.e., a “tree-rings” profile) and avoid lengthy periods of blue sitting. Second, participant step goals were split into three daily periods: *morning*, which fell before noon; *midday* which fell between noon and 5:30 pm; and *night*, which fell after 5:30 pm. During each of these periods, participants could earn up to 45% of their daily stepping goal. If a participant achieved >45%, they would receive a “mastery badge” [i.e., highly specific badge designed to cue the participant to recognize successes (13)] and excess steps would not count toward the daily goal. As such, participants had to move a minimum of 10% during each period to achieve their daily goal. The second key purpose of the Companion App was to provide a sense of connection to the program staff between weekly appointments. Participants received access to weekly educational podcasts on pain, weight, and activity recorded by one study researcher (JF). They received weekly animated videos that reinforced weekly educational content, and they also received regular messages within a “newsfeed” feature from the behavioral interventionist, which could also be used by participants to communicate with their cohort.

Participants were mailed the ActivPAL™ 4 activity monitor that was to be worn during the 12th week of the program. All other assessments were completed after the final week of the program. After completion of all follow-up testing, control group participants who were interested were given the opportunity to receive the MORPH intervention as incentive for participating in the study.

### Measures

#### Pain

Pain intensity was assessed using the PROMIS 3-item pain intensity scale (28). This scale asks participants to reflect on the previous 7 days and rate how intense their pain was at its worse, how intense was their average pain, and what their present level of pain was. Participants responded to each on a 5-point scale where 1 represented “no pain” and 5 represented “severe pain.” Pain interference was assessed using the PROMIS 8-item pain interference scale. Here, participants reflected on the previous 7 days and rated the extent to which pain interfered with their daily activities. Again, responses were provided on a 5-point scale where 1 represented “not at all” and 5 represented “very much.” For both PROMIS measures, scores were uploaded to the PROMIS scoring system, which provides a t-score (mean = 50, SD = 10) for each participant relative to a nationally representative sample.

#### Physical Function

Physical function was assessed using the short physical performance battery [SPPB (16)]. The SPPB is a measure of lower extremity function consisting of walking speed, balance, and repeated chair stand tasks. Performance on each of these three domains produces a score range from 0–4 for a maximum total score of 12. Higher scores on the SPPB represent better lower extremity function.

#### Weight

Participant body weight (in kg) was assessed using a single calibrated scale prior to the start of the intervention and again following cessation of the program. One height at follow-up was missing, and this was replaced with the participant’s baseline height.

#### Physical Activity

The ActivPAL™ 4 device was selected to capture physical activity and sedentary behaviors. This is a small triaxial accelerometer with inclinometer that is worn on the midline of the thigh for 7 days to provide an accurate assessment of posture—of interest here given the focus on reducing sedentary behavior through frequent movement. The device was adhered to the participant’s leg using a clear adhesive patch, resulting in very low non-wear time. Participants with at least two complete days of data were retained in all analyses. We report here on average daily minutes of sedentary time, average daily postural shifts (i.e., sit-to-stand transitions), and average daily steps. We focus on steps rather than minutes of moderate-to-vigorous physical activity as the ActivPAL™ 4 utilizes stepping cadence to estimate movement intensity, and there are at present no thresholds specific to older adults. Unfortunately, there was a high rate of battery failure, resulting in 10 individuals without valid data across both timepoints. Moreover, a postal delay in Spring 2020 caused 8 individuals to receive and wear their monitors after cessation of the 12th week of the program. Therefore, we present the ActivPAL data alongside the Fitbit stepping data, as these were collected serially across the program, which allowed for us to assure data were collected within appropriate windows.

Participants aimed to wear the Fitbit Alta device throughout the study period. This is a commercial grade triaxial accelerometer that is worn on the wrist and designed to capture stepping behaviors. All MORPH study participants were allowed to keep their Fitbit monitors as an incentive for their participation. We collected data via the Fitbit API in one-minute increments to allow for the provision of in-app feedback. To ensure data integrity, we also exported all minute-level Fitbit data from Fitbit servers upon completion of the study for use in analyses. Minute-level stepping data were summarized at the daily level (i.e., total daily steps, minutes of activity measured, breaks from inactive-to-active states), and days with at least 600 min were considered valid. Individuals with two valid days of data were retained in analyses. The reader should be cautioned that Fitbit does not disclose their method for determining valid wear periods. Notably, one control participant failed to upload data to Fitbit during the week prior to the start of the intervention, so the participant’s first-week data were used instead given these participants did not receive any active intervention. Average daily steps and daily breaks were computed for all average days at baseline and during the 12th week of the study. Finally, we would like to note that key sources of missing data we encountered for this monitor includes device failure (e.g., battery failure, Bluetooth pairing failure), and user error (e.g., placing the device on the charger and then forgetting to wear it, losing the device, failing to charge the device).

#### Participant and Interventionist Feedback

To identify potential protocol modifications that may not present in other data sources, the behavioral interventionist queried participants weekly for input on aspects the participant perceived worked well and on things that would benefit from modification. Additionally, the interventionist and nutritionist convened regularly across the intervention period to discuss challenges and successes. All feedback was logged by the behavior interventionist as it was received.

### Analyses

Participant characteristics were summarized using mean ± SD for continuous measures and n (%) for count measures. Given the structure and purpose of phase 1 (i.e., to provide simple, pragmatic feedback to guide app refinement), key changes from each iteration were noted and then briefly summarized by general theme. For phase 2, the effect of the intervention on pain, physical function, physical activity, and weight were each assessed via analysis of covariance (ANCOVA) models wherein follow-up values for each focal variable were included as the dependent variable, group assignment was included as the independent variable of interest, and age, gender, and the baseline value of the focal variable were included as covariates. Given the small sample in this pilot study, we focused on partial *η*^2^ effect sizes from the ANCOVAs following Cohen’s guidelines [(29), p. 285-288] such that *η*^2^ = 0.01 suggests a small effect, *η*^2^ = 0.06 suggests a medium effect, and *η*^2^ = 0.14 suggests a large effect. Each model was checked for the following assumptions: normality of residuals, linearity, homogeneity of regression slopes, homogeneity of variance, homoscedasticity, and for outliers.

## RESULTS

### Phase 1: Refinement

#### Participants

We interviewed five older adults with chronic pain during this phase to reach our phase 1 goal of two consecutive participations without imperative changes. Participant characteristics are displayed in Table 1. All participants were white, and 4 of 5 were male. Average age was 71.8 years. All participants reported hip pain, 4 reported back pain, 4 reported knee pain, 2 reported neck pain, and 2 reported shoulder pain. The sample was similar to the national average (a PROMIS *t* score of 50) on pain intensity but was higher than average for pain interference. Following the one-week field test of the app in the user’s home, the average System Usability Scale score was good-to-excellent, as indicated by an average usability score of 79.0 (30).

#### Key Modifications

The lessons learned from the first phase of the study fell into three broad categories. The first category was **technological tool selection.** The Fitbit Alta device was received well by participants. We were able to easily collect and provide automatic feedback from minute-level activity data via the Fitbit API, and participants found the device easy to use. We initially selected the Fitbit Aria 2 smart weight scale in order to leverage the same API, but found mixed success connecting the Aria 2 to study staff Wi-Fi (a requirement for use). Two of the first three participants also could not connect to their in-home Wi-Fi router. Therefore, we pivoted to the BodyTrace cellular scale, which was utilized across all remaining MORPH participants. Because this scale relied on a cellular chip, participants were instructed to keep the scale along the outer perimeter of the house. No issues were reported using this scale among study team members or in the final two participants.

The second category was **form**; a key design consideration given that older adults who are non-expert in the use of smartphone devices may not find common interface elements intuitive. Participant feedback led to modifications to an in-app group chat feature to clarify interface elements. For instance, the text entry box did not have a dark border and participants did not think to interact with it, so a dark border was added to make it clearer. We also modified phrasing: the title “Enter your Thoughts” was changed to “Chat with your Group,” a button saying “Add to Group” was changed to “Post to Group” to aid in clarity. Participants also wanted to know where data came from, and so a note was added to the Data Viewer to clarify that the data came from the Fitbit device, noting the time of last synchronization. In general, these modifications highlighted the importance of overt labeling of in-app features when working with this population.

The final category was **clarity of purpose**. Two participants had trouble conceptualizing the day-long movement recommendation and the role of the in-app feedback for supporting this recommendation, based upon the single brief orientation appointment. Conversely, three participants reported the app infrastructure was easy to interpret, and found they enjoyed moving throughout the day. Participant two noted: “my back is worse when I sit more,” while participant four noted “the long blue spans [on my feedback bar] meant I was sitting too long and needed to get up and move.” To attempt to address this heterogeneity in understanding during the second phase of MORPH, we provided participants with an animated video on the importance of moving throughout the day and included didactic content in early group sessions on this topic.

### Phase 2: Pilot

#### Descriptive Information and App Usage

**FIGURE 2 ∣:**
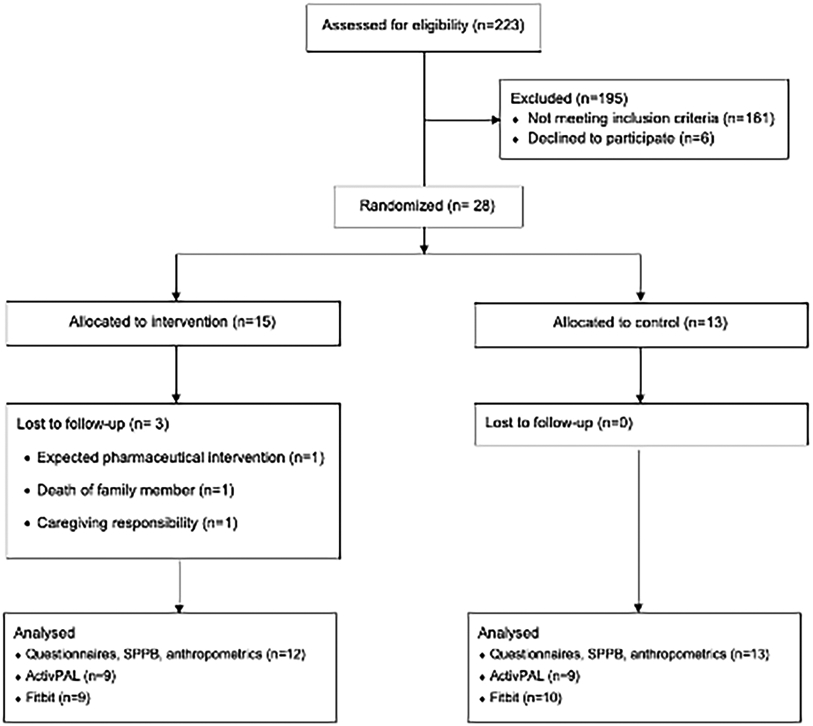
Consort Diagram. SPPB, short physical performance battery.

**FIGURE 3 ∣:**
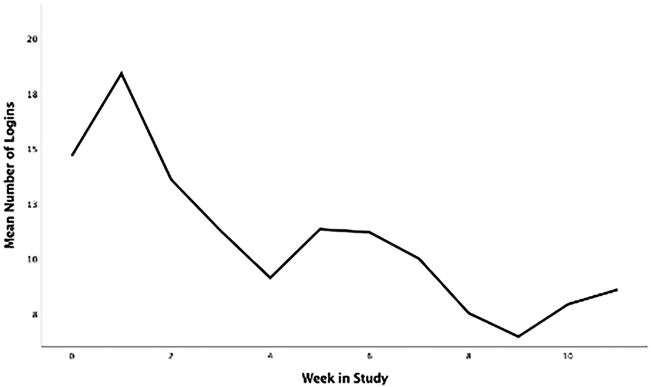
Average weekly app uses (opening app from home screen) in the intervention condition over the 12-week study.

Participant characteristics for phase 2 are displayed in Table 2, and the CONSORT diagram is provided in Figure 2. In total, 223 participants were screened, and 28 participants were eligible and agreed to participate (15 intervention; 70.21 ± 5.22 years). Briefly, participants had an average SPPB score of 9.61 ± 1.71 out of 12 at baseline, and an average BMI of 36.96 ± 4.54 kg/m^2^. Twenty-two (78.6%) were female, 23 (82.1%) were white, 24 (85.7%) had at least a college degree, and 100% of control participants and 12 of 15 intervention participants (80.0%) completed follow-up testing. Average participant app usage (i.e., average weekly accesses) are depicted in Figure 1. This captures the count of times a participant opened the app from the device’s home screen. Unique daily application accesses remained high throughout the study period, demonstrating a small and expected decrease over 12 weeks, generally remaining above an average of one daily access throughout the study duration. The highest weekly average accesses were in the second week (18.40) and the lowest were in the ninth week (6.47). Average weekly app uses are depicted in Figure 3.

#### Results From Analyses of Covariance

Week 12 means, adjusted for baseline values for each dependent variable, sex, and age are provided in Table 3. The ANCOVA for physical function, which controlled for age, gender, and baseline SPPB score, revealed a moderate effect size in favor of the intervention condition, *F*_(1,20)_ = 1.675, *p* = 0.210, *η*^2^ = 0.08. This corresponded to an adjusted difference of 0.63 points, which is within with range of a “substantial” clinically-meaningful difference [0.4–1.5 (31)].

Regarding pain interference, there was one outlying residual (i.e., Z > 3) in the intervention condition. This was addressed by winsorizing the participant’s week 12 score to within 3 standard deviations. The ANCOVA for pain interference suggested a small effect, *F*_(1,20)_ = 0.610, *p*= 0.444, *η*^2^ = 0.03. The adjusted interference score was 1.60 points higher in the intervention condition; a difference below the minimally important difference identified for fixed-length PROMIS pain intensity measures collected in individuals with chronic pain [2.0–3.0 points (32)]. There was a moderate-to-large effect for pain intensity favoring the intervention condition, *F*_(1,20)_ = 2.752, *p* = 0.113, *η*^2^ = 0.12. This corresponds with an adjusted difference at follow-up of 5.52 points. Although there are no guidelines for clinically meaningful difference in pain intensity among older adults, this corresponds to a more than half-standard deviation difference between groups.

The ANCOVA for body weight suggested a large and statistically significant effect in favor of the intervention condition, *F*_(1,20)_ = 5.233, *p* = 0.033, *η*^2^ = 0.207, corresponding to a 2.90 kg lower adjusted weight at follow-up in the intervention condition. Notably, unadjusted weight in the intervention condition reduced from 97.62 kg at baseline to 94.83 kg at follow-up, reflecting a weight loss of approximately 3% (i.e., the study’s targeted weight loss).

In total, 10 control participants and 9 intervention participants had at least 2 valid days of Fitbit data at both timepoints. The ANCOVA revealed a moderate-to-large effect on average daily Fitbit-recorded steps favoring the intervention group, *F*_(1,14)_ = 1.726, *p* = 0.210, *η*^2^ = 0.11. At follow-up, intervention participants had 756.55 more daily steps compared to control after controlling for baseline. There was a moderate effect on Fitbit-assessed breaks that favored the control condition, *F*_(1,14)_ = 0.885, *p* = 0.363, *η*^2^ = 0.06 such that control participants obtained approximately 5 additional daily breaks at follow-up. In total, 9 control and 9 intervention participants had at least two valid days of ActivPAL data at both timepoints. There was a moderate effect on ActivPAL-assessed sedentary time, *F*_(1,13)_ = 0.959, *p* = 0.345, *η*^2^ = 0.07, such that intervention participants sat for 64.90 min less daily at follow-up compared to control participants after adjusting for baseline values, sex, and age. There was a small effect on ActivPAL™-recorded daily steps, *F*_(1,13)_ = 0.145, *p* = 0.709, *η*^2^ = 0.01, such that those in the intervention condition achieved 297.70 additional adjusted daily steps relative to those in the control condition.

#### Participant and Interventionist Feedback

Phase 2 lessons learned fell into two themes: technology and participant coaching. Regarding technology, a key challenge encountered in the conduct of MORPH was in the teleconferencing software selected for remote delivery, which was selected due to local institutional support. The software favored the most active audio feed—which often included background noise in participant homes such as a loud television set—while muting less-active feeds. This necessitated the interventionist muting participants by default and unmuting when a participant signaled a desire to speak; a strong limitation in the context of a group-mediated intervention. Additionally, participants and the interventionist alike noted the flattened nature of communicating through a two-dimensional medium that lacked the sensation of physical proximity and presence. Finally, there was a fairly high rate of device failure across both activity monitors. We purchased all Fitbit and ActivPAL devices early in the MORPH study period, up to a year and a half prior to the start of phase 2 of the study, and so the long period without use may have contribute to connectivity and battery issues we observed. While there are no published statistics on failure rates for these devices, poor battery longevity is a common topic in Fitbit user forums—a search for “Alta Battery” on the Fitbit website produces more than 2,000 results primarily pertaining to rapidly draining batteries—and has been cited as a common limitation of the devices in popular press (33). We also had a high rate of battery failure within our research grade monitor (i.e., the ActivPAL™), underscoring the importance of conducting regular battery health screenings when using accelerometers.

Regarding coaching, it was clear that many participants gravitated toward the weight loss portion of the study and tended to focus on traditional modes of exercise (e.g., extended daily walks) rather than day-long movement. This is supported by the absence of significant change in distribution of activity (e.g., sedentary breaks). There were greater steps and fewer minutes spent sitting in the intervention condition, suggesting these individuals tended to engage in a small number of relatively longer bouts of movement. It is likely that more intensive coaching is needed especially early in the adoption phase of day-long movement. Additionally, the interventionist noted the power of pain as a daily barrier to movement. In one instance, a participant reported deciding on whether to leave their bed upon waking each morning, depending on their pain. This represents an immensely challenging intervention target: how does one intervene in these critical moments?

## DISCUSSION

Our aims were to identify and address and use and usability issues in the MORPH app and to examine the feasibility of delivering the MORPH intervention in older adults with chronic pain and to evaluate the potential impact of the MORPH intervention on physical function, body weight, pain, and physical activity. We identified several limitations in our refinement phase that would have impaired uptake of the app if left unaddressed, with the most pressing including a smart scale that rarely functioned in the field (the Fitbit Aria 2), and lack of clarity on several interface elements. This underscores the importance of conducting and disseminating refinement testing within mHealth interventions. Additionally, based on strong rates of application usage over time and positive effects on pain, we believe the pilot phase provides strong evidence for the feasibility of a home-based and technology-supported behavioral intervention for older adults with chronic pain. In addition to positive effects on pain intensity, we observed clinically meaningful effects on physical function, a reduction in body weight of approximately 3%, a moderate increase in steps, and more than 1 hour less sitting each day. These early findings are promising for several reasons and, when paired with feedback gleaned in the conduct of the study, provide preliminary support for the merits of treating chronic pain in older adults with obesity via remotely delivered health behavior interventions.

First, MORPH relied heavily upon technology for its delivery, including the use of several smart devices (i.e., Fitbit Alta, BodyTrace Scale), several smartphone applications, and video conferencing software. These early findings suggest this is a feasible method for delivering behavioral interventions to older adults. Generally, participants accessed the app approximately once daily across the study. Expectedly, daily accesses were higher early in the program as participants enjoyed the novelty of the tools, spent time exploring the interface, and worked to internalize skills such as self-monitoring of movement across the day. As Ritterband (34) notes, digital health tool usage can be expected to decrease as individuals complete discrete tasks (e.g., watching a video) or internalize skills taught by the toolset (e.g., self-monitoring movement patterns). Conversely, declining use may also signal waning interest in the study self-monitoring tools. It will be important to determine how trajectories of change in mHealth tool use influence behavioral and health outcomes over longer timescales in future iterations of this program. There were also several technology-related challenges that emerged during the RCT phase of MORPH: The Fitbit Alta was easily worn by participants, however, we experienced high rates of missing data resulting from both the Fitbit and ActivPAL™ devices. As such, regular battery health monitoring is a must. The video conferencing software we selected did not allow for comfortable conversation and did not provide the sense of physical proximity that is important for the development of social bonds. Indeed, it appears that physical proximity tends to be key to developing interpersonal liking (35). Thus, a priority for future iterations of MORPH will be the identification of communication media that can provide a better sense of physical presence. One particularly exciting possibility lies in recent developments in virtual reality, including units that are fully self-contained (i.e., requiring no connection to a powerful external computer). As this technology advances, devices become easier to use relative to previous generations, allow the user to navigate using their own hands rather than via input devices, are designed explicitly to produce feelings of immersion and physical presence (36), and include onboard microphones and speakers that together allow for rich, natural vocal communication. Virtual reality uptake is rising rapidly, with the global VR market expected to grow from $5.34 billion in 2017 to $56.25 billion in 2025 (37). As such, it is imperative that the safety and utility of the devices is established among older adults, and evidence-based recommendations are crafted to guide the development of effective behavioral interventions.

Beyond the technology employed in MORPH, we found that an intervention that was primarily delivered remotely can produce weight loss and improvements in both physical function and pain intensity in older adults with chronic pain. Fortunately, the dietary weight loss and pain management strategies we employed are well-suited for remote delivery, which suggests these are good candidates for use in a large-scale and fully remote randomized trial. As noted previously, our weight loss protocol includes simple self-monitoring via daily food logs plus in-app feedback on body weight as collected via smart scale. It uses group-mediated behavioral counseling and simple-to-understand weekly macronutrient goals intended to produce a modest weekly weight loss. To maintain this important group structure, sustainable methods for delivering in-home group-mediated counseling are needed to produce lasting dietary behavior change. Fortunately, effective online therapy platforms are seeing greater uptake (38), including online group therapy, and this represents one promising avenue for perpetual delivery of group health behavior change protocols.

While we identified several components that will be retained in future iterations of MORPH, including the group-mediated dietary intervention and general remote delivery structure, we also identified several components that we will continue to optimize. In addition to the application of strong quality control over activity monitors and the identification of presence-promoting communication media, it is clear that the day-long physical activity prescription requires more intensive coaching early in the study. Specifically, it is important that interventionists help participants break preconceived notions of what constitutes health-enhancing activity (i.e., shifting focus away from single bouts of structured exercise) and more strongly emphasize the importance of frequent bouts of movement to disrupt sitting. Lastly, this pilot was focused on refining and testing the technology-supported protocol, and so measures of social connectivity were not collected. In our next iteration of MORPH, we intend to assess effects on social connection and test the impact of varying contact schedules on uptake of the day-long movement intervention as reflected in daily steps, daily sit-to-stand transitions, and average sedentary bout length.

The MORPH randomized controlled trial had several notable strengths, including the delivery of a robust socially mediated intervention to older adults in the home via technology, positive effects across a host of key health outcomes, and the use of an iterative design that fosters optimization of the intervention package as well as publication of key successes and challenges to guide health promotion experts and technology developers. There were also several important limitations beyond the challenges described above. Chiefly, we recruited a small pilot sample comprised of participants that were generally well-educated and white, and a majority of our sample were female, though this expected as the majority of chronic pain sufferers are women (39). Approximately 82% of our sample were White, compared with approximately 72% nationally, and the remaining 12% were Black, compared with approximately 10% nationally. No individuals identifying as Hispanic were recruited, though within the United States 12% of those with chronic pain identify as Hispanic (40). As these demographic factors may affect perceived usefulness and usability, future iterations will aim to recruit larger samples with better representation across race and education. Next, we did not observe a meaningful difference in the extent to which pain interfered in individuals’ daily life. It may be that a longer intervention period, allowing for greater weight loss, may be required to improve pain interference (41). Given the purpose of this pilot study (i.e., application refinement, establishing feasibility of the MORPH program), we had limited statistical power. A fully powered clinical trial will be an important next step in supporting the utility of this potentially scalable approach to weight loss and pain management. Likewise, given the phasic structure wherein the first phase focused on application refinement and the second phase focused on feasibility and initial effects of the MORPH intervention, we did not get usability data during the second phase. It will be valuable to continue to collect usability data in future iterations as needs and preferences are likely to evolve as communication technologies continue to evolve. Still, we believe our results show consistent and positive effects in favor of our intervention and provide useful lessons to those interested in developing group-mediated, home-delivered behavior change programs for older adults.

## CONCLUSIONS

Chronic pain is prevalent in aging and functions to reduce physical activity, impair physical functioning, and to produce weight gain. MORPH represents an important step in the design of an accessible and lasting weight loss and activity intervention for older adults with chronic pain. These findings suggest there is value in a group-mediated model designed for in-home delivery using concepts drawn from social cognitive and self-determination theory. In the spirit of multiphase optimization, we are at present collecting qualitative focus group and interview data on former MORPH participants to refine our understanding of program features participants viewed as helpful and burdensome. These data, alongside the results presented herein, will be used to refine our approach to promoting movement across the day. Ultimate success in this endeavor will provide a useful model for future commercially delivered programs that can facilitate long-term group behavioral counseling and in turn lasting behavior change.

## Supplementary Material

Supplementary Table

## Figures and Tables

**TABLE 1:** Participant Characteristics within the Iterative Refinement Phase (N=5)

Gender	
Male (N, %)	4, 80
Female (N, %)	1, 20
Race	
White	5, 100%
Age (mean ± SD)	71.8 ± 5.54
BMI	35.84 ± 2.51
Pain location (N)	
Back	4
Neck	2
Shoulder	2
Hip	5
Knee	4
PROMIS Pain Intensity (t score±SD)	50.44±1.85
PROMIS Pain Interference (t score±SD)	57.32±2.87
SUS Total Score (mean±SD)	79.00±21.33

Notes: BMI = Body mass index, kg/m^2^; PROMIS = Patient-Reported Outcomes Measurement Information System; SUS = System usability scale.

**TABLE 2.** Participant Characteristics at Baseline within the Pilot Phase

	Intervention (n = 15)	Control (n = 13)	Overall (N = 28)
Age (years; M±SD)	70.12±5.43	70.32±5.20	70.21±5.22
Sex (n,%)			
Female	13 (86.7)	9 (69.2)	22 (78.6)
Male	2 (13.3)	4 (30.8)	6 (21.4)
Race (n,%)			
Black	3 (20)	2 (15.4)	5 (17.9)
White	12 (80)	11 (84.6)	23 (82.1)
Education (n,%)			
High School Graduate	2 (13.3)	2 (15.4)	4 (14.3)
College Graduate	9 (60.0)	7 (53.8)	16 (57.1)
Post-Graduate	4 (26.7)	4 (30.8)	8 (28.6)
Weight (kg±SD)	98.41±5.88	98.91±18.22	98.64±16.68
BMI (kg/m^2^; M±SD)	36.9±4.84	36.92±4.36	36.96±4.54
SPPB (M±SD)	9.73±1.28	9.46±2.15	9.61±1.71
PROMIS 8-item Pain Interference (t-score±SD)	59.92±8.59	58.80±7.74	59.40±8.08
PROMIS 3-item Pain Intensity (t-score±SD)	64.21±3.85	60.42±6.12	62.45±5.30

Notes: M = mean; SD = standard deviation; BMI = body mass index; SPPB = short physical performance battery, score out of 12; PROMIS = patient-reported outcomes measurement information system);

**TABLE 3.** Adjusted Means at Week 12 (Mean±SE).

	Intervention (n = 12)	Control (n = 13)
SPPB	10.20±0.346	9.580±0.333
PROMIS 8-item Pain Interference (t-score±SD)	63.269±1.470	61.667±1.411
PROMIS 3-item Pain Intensity (t-score±SD)	54.270±2.329	59.789±2.232
Weight (kg)	95.377±0.907	98.275±.871
Fitbit Steps*	3422.128±416.898	2665.579±395.418
Fitbit Breaks*	52.529±4.578	58.558±4.335
AP Steps**	4402.625±518.339	4104.931±518.339
AP Sitting Time (m)**	651.108±44.579	716.010±44.579

All means are adjusted for baseline values, age, and participant sex. *Notes*: SPPB = short physical performance battery, score out of 12; PROMIS = patient-reported outcomes measurement information system); AP = ActivPAL

* n=9 intervention, n=10 control

** n=9 intervention, n=9 control

## Data Availability

The raw data supporting the conclusions of this article will be made available by the authors, without undue reservation.
