# Supplementary material for: A Mobile Health Behavior Intervention to Reduce Pain and Improve Health in Older Adults With Obesity and Chronic Pain: The MORPH Pilot Trial
Source: Front Digit Health. 2020 Dec 18;2:598456. doi: 10.3389/fdgth.2020.598456 (PMC8018691; doi:10.3389/fdgth.2020.598456)
Supplement: Supplementary file 1 [file Table_1.DOCX]

Supplementary Material

# Supplementary Tables

Supplemental Table 1: Focal App Elements

| 1. Earned-badge Alert | Displays when the app is launched, letting the user know they have earned a movement badge. User is to tap a button to close the alert. |
| --- | --- |
| 1. Home Button | Returns the user to the home screen |
| 1. Data Button | Displays digested Fitbit data |
| 1. News Button | Displays social feed |
| 1. Date Button | Allows participant to view retrospective data |
| 1. Timeline Bar | On Home screen, provides graphic visualization of movement (green) and sitting (blue). When touched triggers a window containing the time of day and whether the person was sitting or moving during that time. |
| 1. Badge Button | On Home screen, when touched opens collapsed “badge bin” which holds earned badges. |
| 1. Individual Badge | Within “badge bin”, touching a badge displays a pop-out describing what the badge rewards. |
| 1. Weight Quick-view | On Home screen, a small bar displaying most recent weight. |
| 1. Steps chart | On Home screen, a pie chart displaying *periodic* steps. Also notes when at least 1 daily period is maximized. |
| 1. Breaks chart | On Home screen, displays number of times the user went from a movement minute to sedentary minute on the Fitbit. |
| 1. Dr. Amber’s Resources | On Home screen, user taps Dr. Amber to view current resources (videos, podcasts). |
| 1. Data Picker | When the Data Button is pressed, the Data Picker is displayed. User is expected to explore different Fitbit-derived metrics and to note the option to refresh Fitbit data manually and to log out. |
| 1. News Feed | When the News Button is pressed, the News Feed is displayed. The user is expected to note pre-populated messages from the research team or other study members. They are expected to submit a new comment and to notice options to edit and delete their post. |
| 1. Date Picker | When the Date Button is pressed a date picker is shown. The user is expect to alter the date and recognize that this produces data from the selected date. |
