# Supplementary material for: A Mobile Health Behavior Intervention to Reduce Pain and Improve Health in Older Adults With Obesity and Chronic Pain: The MORPH Pilot Trial
Source: Front Digit Health. Author manuscript; Available in PMC 2021 Apr 2. (PMC8018691; doi:10.3389/fdgth.2020.598456)
Supplement: Supplementary file 1 [file NIHMS1685333-supplement-Supplementary_Table.pdf]

Supplementary Material

1 Supplementary Tables

Supplemental Table 1: Focal App Elements

1. Earned-badge Alert	Displays when the app is launched, letting the user know they have earned a movement badge. User is to tap a button to close the alert.
2. Home Button	Returns the user to the home screen
3. Data Button	Displays digested Fitbit data
4. News Button	Displays social feed
5. Date Button	Allows participant to view retrospective data
6. Timeline Bar	On Home screen, provides graphic visualization of movement (green) and sitting (blue). When touched triggers a window containing the time of day and whether the person was sitting or moving during that time.
7. Badge Button	On Home screen, when touched opens collapsed “badge bin” which holds earned badges.
8. Individual Badge	Within “badge bin”, touching a badge displays a pop-out describing what the badge rewards.
9. Weight Quick-view	On Home screen, a small bar displaying most recent weight.
10. Steps chart	On Home screen, a pie chart displaying *periodic* steps. Also notes when at least 1 daily period is maximized.

11. Breaks chart	On Home screen, displays number of times the user went from a movement minute to sedentary minute on the Fitbit.
12. Dr. Amber's Resources	On Home screen, user taps Dr. Amber to view current resources (videos, podcasts).
13. Data Picker	When the Data Button is pressed, the Data Picker is displayed. User is expected to explore different Fitbit-derived metrics and to note the option to refresh Fitbit data manually and to log out.
14. News Feed	When the News Button is pressed, the News Feed is displayed. The user is expected to note pre-populated messages from the research team or other study members. They are expected to submit a new comment and to notice options to edit and delete their post.
15. Date Picker	When the Date Button is pressed a date picker is shown. The user is expect to alter the date and recognize that this produces data from the selected date.
